# Surgical resection for hepatocellular carcinoma with portal vein tumor thrombus in the Asia-Pacific region beyond the Barcelona Clinic Liver Cancer treatment algorithms: a review and update

**DOI:** 10.18632/oncotarget.18735

**Published:** 2017-06-27

**Authors:** Jia-Zhou Ye, Yan-Yan Wang, Tao Bai, Jie Chen, Bang-De Xiang, Fei-Xiang Wu, Le-Qun Li

**Affiliations:** ^1^ Department of Hepatobiliary Surgery, Affiliated Tumor Hospital of Guangxi Medical University, Nanning 530021, PR China

**Keywords:** hepatocellular carcinoma, portal vein tumor thrombus, surgical resection

## Abstract

Portal vein tumor thrombus (PVTT) usually worsens prognosis of hepatocellular carcinoma (HCC), as characterized by aggressive disease progression, impaired liver function and tolerance to treatment. Conventionally, the European Association for the Study of the Liver (EASL) and the American Association for the Study of Liver Diseases (AASLD) accepted the Barcelona Clinical Liver Cancer (BCLC) treatment algorithms, identifying PVTT as an absolute contra-indication of surgical resection for HCC. HCC-PVTT patients are offered sorafenib as the standard treatment. Evidently, SHARP and Asia-Pacific trials demonstrated that sorafenib only improves overall survival by approximately 3 months in patients with advanced HCC. Besides, BCLC treatment algorithm does not provide different therapeutic recommendations for different degree of PVTT, and only supports single treatment option for each stage of HCC rather than a combination of comprehensive treatments, which limited individual and best care for every HCC-PVTT patients. In the past few years, many surgeons do not restrict surgical resection to HCC with PVTT. There have been new reports demonstrated that surgical treatment is feasible for selected HCC-PVTT patients with resectable tumor and moderate liver function to prolong survival period and elevate life quality as long as PVTT limited to the first-order branch, whereas non-surgical treatments fail to provide comparable therapeutic effects. At present, guidelines on HCC management from mainland China, Japan, and Hong Kong have been updated and a consensus of Asia-Pacific experts has established that portal venous invasion is not an absolute contradiction of surgical resection for HCC. This review summarized the emerging data on surgical resection for HCC-PVTT patients beyond the BCLC treatment algorithms and discussed recent therapeutic conceptualchanges in the Asia-Pacific region.

Hepatocellular carcinoma (HCC) is the sixth most common cancer and one of the most prevalent leading causes of cancer-related death worldwide [1]. Owing to the biological characteristics of HCC and the anatomical characteristics of the liver, HCC is prone to invade intrahepatic vessels, especially the portal venous system. Portal vein tumor thrombus (PVTT) was detected in about 10–60% of patients with HCC at the time of diagnosis, [2] and has been proved to play an important role in prognosis and clinical staging of HCC [3–4]. Once the PVTT has developed rapidly and progressed into the contralateral bifurcation or the main trunk of portal vein, obstruction by the tumor thrombus usually promotes disease progression, aggravates portal vein hypertension and its related complications, deteriorates liver function reserve, and induces tolerance to anti-tumor treatment. Moreover, when the primary tumor invades the portal venous system, HCC cells become distributed along the branches of the portal vein and spread to adjacent liver segments, leading to invisible intrahepatic metastasis, which has been well accepted as a major mechanism contributing to early intrahepatic recurrence [5–9]. The prognosis of patients with HCC and PVTT is extremely poor: the median survival period is only 2.7–4 months, as compared with 10–24 months in patients without PVTT [10]. Conventionally, Western associations, such as the European Association for the Study of the Liver (EASL) [11] and the American Association for the Study of Liver Disease (AASLD) [12] accepted the Barcelona Clinic Liver Cancer (BCLC) staging and treatment algorithm for the management of HCC, which indicates PVTT as an absolute contra-indication for surgical resection, owing to the substantial risks of insufficient remnant liver function reserve and tumor recurrence. Instead, the BCLC treatment algorithm only recommends sorafenib as the standard treatment [11–12].

Evidently, two phase III randomized, double-blind, placebo-controlled trials, SHARPE trial (in Europe and USA) [11] and Asian-Pacific study (in the Asia-Pacific regions), [12] demonstrated unpromising outcomes; sorafenib only prolongs the overall survival period by approximately 3 months in patients with advanced HCC. Moreover, owing to the BCLC treatment algorithm does not provide different therapeutic recommendations for different degree of PVTT, and only supports single treatment option for each stage of HCC rather than a combination of comprehensive treatments, which limited BCLC treatment algorithm to provide the individual and best care for every HCC-PVTT patients. In practice, many surgeons do not restrict surgical resection to HCC with PVTT. In the past years, many studies [13–49] reported that selected patients with HCC and PVTT were treated by surgical resection beyond the BCLC treatment algorithm worldwide, especially in the Asia-Pacific region. These studies [13–49] approved surgical resection as an effective treatment option for selected patients with HCC and PVTT to prolong the survival period and enhance life quality. Moreover, the developments of advanced multidisciplinary diagnoses and treatments have greatly improved the safety and efficacy of surgical resection. Such management includes accurate preoperative assessments of anatomic characteristics of the primary liver tumor and PVTT, prediction of residual functional liver volume, preoperative radiotherapy to downstage the primary tumor and PVTT, and postoperative transarterial chemoembolization (TACE) to suppress and monitor early tumor recurrence. Based on new evidence, some guidelines from mainland China, [50] Hong Kong, [51] and Japan [52] have been updated and a consensus of Asian experts [53] has established that surgical resection may be considered in selected patients with HCC and PVTT to prolong the survival period and improve life quality, even where a complete cure for this malignant disease is not possible.

This paper summarizes new evidence on surgical resection and advanced multidisciplinary diagnosis and adjuvant treatments for patients with HCC and PVTT. We also discuss recent conceptual changes beyond the BCLC treatment algorithm in the Asia-Pacific region.

## METHODS

A computerized literature search of Medline and EMBASE was performed, using the advanced search model and the following combinations of search terms: “hepatocellular carcinoma” *or* “hepatoma” *or* liver cancer” *or* “liver tumour” *or* “liver neoplasm” *and* “portal vein tumor thrombus” *or* “advanced stage” *or* “BCLC-C stage” *or* “portal vein invasion” *and* “resection” *or* “hepatectomy” *or* “surgery.” Only English-language articles were analyzed. Studies were included in our review if they evaluated the efficacy of surgical resection to treat HCC with PVTT and included data on at least one of the outcomes of media survival, overall survival, disease-free survival, postoperative complications, or in-hospital mortality. The last search update was made in December 2016. Additional citations were searched manually. Data were extracted directly from tables or the text whenever possible and analyzed by 4 authors. If the data were presented only in graphs, they were extracted by manual interpolation. For the included studies, 1, 3, and 5 year overall survival and postoperative complications were summarized graphically using bubble plots, [54] in which relative sample size was proportional to bubble size. In addition, changes in 1, 3, and 5 year overall survival and postoperative complications over time were analyzed using least-squares weighted regression according to sample size.

## PVTT CLASSIFICATIONS

The HCC staging systems commonly used today are the TNM staging, BCLC staging, and Japanese integrated staging (JIS) systems. All of these HCC staging systems accept the presence of PVTT as closely linked to the prognosis of HCC. However, they do not further define the location and extent of PVTT. At present, two PVTT classifications further define the anatomic characteristics of portal vein invasion and the extension of PVTT: Cheng's classification [55] and the Japanese VP classification [56]. A comparison between the two PVTT classifications is summarized in Table 1. These current PVTT classifications are meaningful in evaluating the disease progression, guiding the choice of therapeutic strategy, and determining the prognosis of patients with HCC and PVTT. Hence, they have been well accepted and widely used in the Asia-Pacific region.

**Table 1:** Classifications of PVTT

Cheng's classification	VP classification	Surgical methods
**Type I0:** Tumor thrombi formation found under microscopy		hepatectomy
**Type I:** Tumor thrombi involving segmental branches of portal vein or above **Type Ia:** Tumor thrombi involving segmental branches of portal vein or above **Type Ib:** Tumor thrombi involving segmental branches of portal vein extending to sectoral branch	**Vp1:** PVTT involving in distal to second-order portal branches **Vp2:** PVTT involving in second-order branches	hepatectomy
**Type II:** Tumor thrombi involving right/left portal Vein **Type IIa:** Tumor thrombi involving right/left portal vein **Type IIb:** Tumor thrombi involving both left and right portal veins	**Vp3:** PVTT involving in first-order branches	hepatectomy
**Type III:** Tumor thrombi involving the main portal vein trunk **Type IIIa:** Tumor thrombi involving the main portal vein trunk for no more than 2 cm below the confluence of the left and right portal veins **Type IIIb:** Tumor thrombi involving the main portal vein trunk for more than 2 cm below the confluence of the left and right portal veins	**Vp4:** PVTT involving in the main portal vein, or contralateral or both	Hepatectomy + thrombectomy or en bloc resection
**Type IV:** Tumor thrombi involving the superior mesenteric vein, or inferior vein cava **Type IVa:** Tumor thrombi involving the superiormesenteric vein **Type IVb:** Tumor thrombi involving the inferior vein cava		Hepatectomy + thrombectomy or en bloc resection

## SURGICAL RESECTION FOR HCC WITH PVTT

### Selected inclusion criteria for patients treated with surgical resection

Based on reports in the literature, patients treated with surgical resection were carefully selected. Inclusive criteria for surgical resection were as follows: (1) good general and medical conditions, with Eastern Cooperative Oncology group (ECOG) scores 0–2; (2) moderate liver function with Child–Pugh class A or B; (3) primary HCC confirmed as resectable without hemi-liver and extrahepatic metastasis; and (4) moderate residual liver function reserve.

### Method of surgical resection

Commonly performed methods and techniques of surgical resection included:

1. Segmental hepatectomy. The PVTT has only invaded the segmental branches of the portal vein where the primary tumor is located [18, 38, 43].
2. Hemi-hepatectomy. The PVTT is only involved in the left portal vein, and the primary tumor is located in the left or right half of the liver without extending beyond the hemi-liver. Based on these 2 anatomic features of the relationship between the primary tumor and PVTT, the PVTT could be removed together with the primary tumor; this method is suitable for Cheng's I–IIa and VP1–3 PVTT types [16, 18, 33, 38, 43].
3. Hepatectomy plus thrombectomy or an embolectomy. These methods are suitable for Cheng's III–IV and VP4 PVTT. When the PVTT extends to the contralateral bifurcation of the left and right portal vein, or the main portal vein, a concomitant thrombectomy is performed with temporary occlusion of portal vein before removal of the PVTT; the PVTT is then extracted through an incision in the portal vein. In addition, an en-bloc resection, including the portal vein bifurcation with or without the main or contralateral portal vein, could be performed if the portal vein branches can be ligated with sufficient and safety margins between the root and the tip of PVTT [16, 18, 33, 38, 43].
4. Portal vein reconstruction followed portal vein resection. When the tumor thrombus invades the main portal vein wall and removal is difficult, the invaded main portal vein might be resected along together with the PVTT in the hepatectomy; a direct end-to-end portal vein anastomosis is then conducted with 6/0 Prolene continuous suture with a 1-cm growth factor [16, 18, 33, 38, 43].

### Efficacy and safety of surgical resection for HCC with any type of PVTT over time

**Figure 1:**
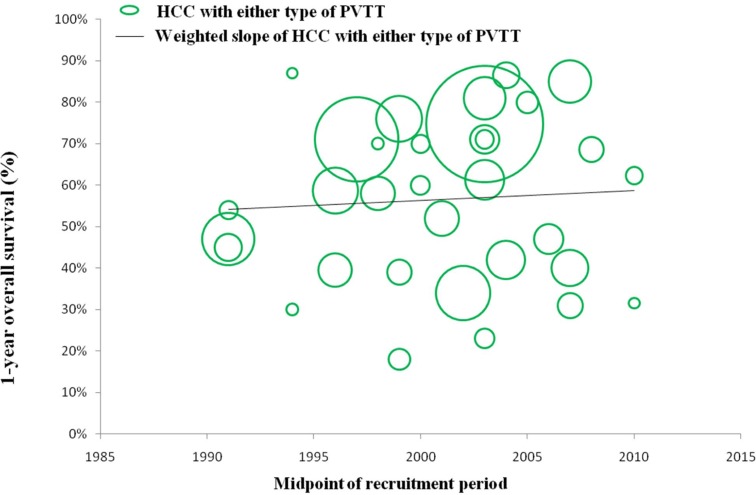
Trend in 1-year overall survival of HCC patients with either type of PVTT

**Figure 2:**
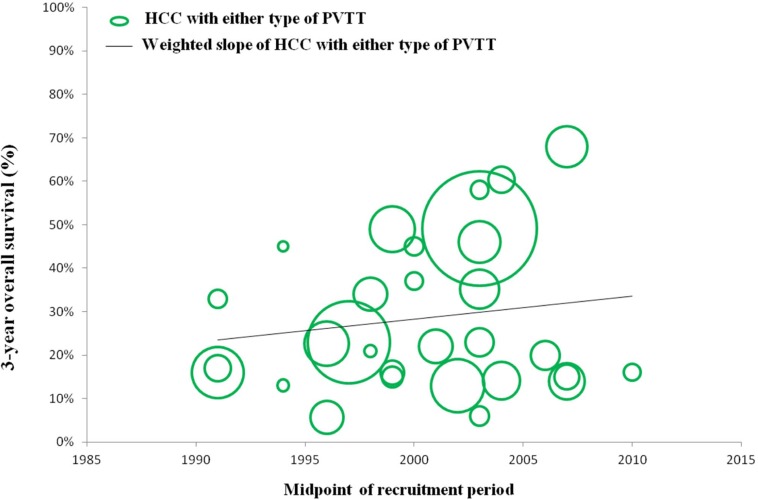
Trend in 3-year overall survival of HCC patients with either type of PVTT

**Figure 3:**
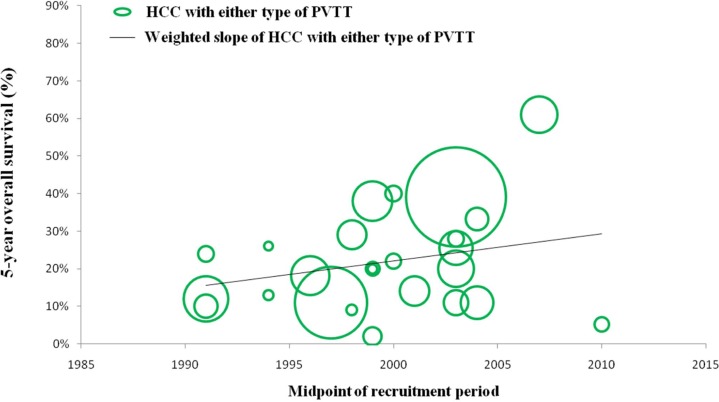
Trend in 5-year overall survival of HCC patients with either type of PVTT

**Figure 4:**
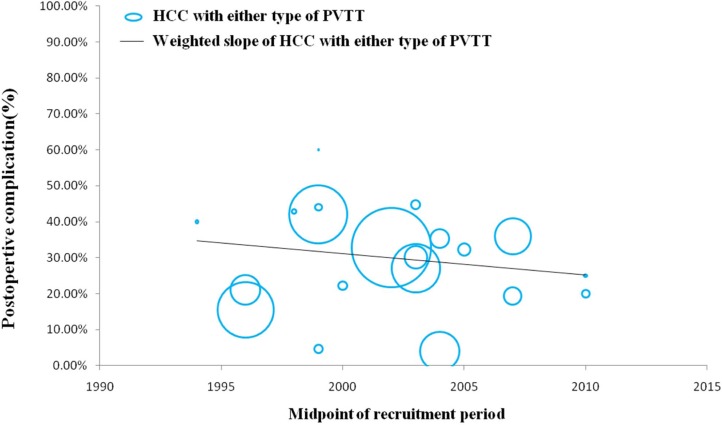
Trend in postoperative complications of HCC patients with either type of PVTT

In a number of reports concerning any type of PVTT, overall survival at 1, 3, and 5 years after surgical resection ranged from 23.4% to 87%, 0% to 68%, and 0% to 61%, respectively. Moreover, disease-free survival at 1, 3, and 5 years after surgical resection ranged from 8.4% to 73.3%, 0% to 32%, and 0% to 22%, respectively. Morbidity and mortality rates across the included studies ranged from 4% to 60% and 0% to 6.25%, respectively. (Studies on surgical resection to treat HCC with PVTT are summarized in Table 2). It is notable that there is a wide range of reported survival rates of HCC-PVTT patients treated with surgical resection due to two major reasons. Firstly, these studies were reported ranged from 1980 to 2012. During the last two decades, surgical techniques and managements have been greatly developed. Thus, we conducted the bubble plot analysis of the studies and we found that when the results were aggregated, the 1-, 3-, and 5-year overall survival periods for patients with HCC and PVTT after surgical resection showed an upward trend (Figures 1, 2, 3), while postoperative complications presented a downward trend over time (Figure 4). The most frequent complications were bleeding, sepsis, abdominal infection, ascites, liver function insufficiency or failure, biliary leakage, and pulmonary complications. Disease-free survival rates and in-hospital mortality did not change appreciably (data not presented). The efficacy and safety of surgical resection for patients with HCC and PVTT have greatly improved in the past few decades, which strongly encouraged surgeons to perform the aggressive approach to treat HCC with PVTT in the Asian-pacific region. Secondly, these studies did not performed stratified analysis based on the PVTT classification. Thus, we consequently analyze the efficacy of surgical resection for HCC-PVTT patients with different degree of PVTT as bellow.

**Table 2:** Postoperative complications, hospital mortality and survival of HCC patients with either type of PVTT treated by surgical resection

Studies	Enrollment period	Total patients	Postoperative complications	Hospital mortality	Median survival period	OS			DFS		
						1-yr	3-yr	5-yr	1-yr	3-yr	5-yr
Zhou [44]	1980-2002	381	-	-	Ca 9 months	47%	16%	12%	-	-	-
Pawlik [25]	1984-1999	102	-	5.9%	11 months	45%	17%	10%	-	-	-
Ohkudo [24]	1985-1997	47	-	2.1%	-	54%	33%	24%	31%	18%	-
Poon [28]	1989-2000	20	-	5%	6 months	30%	13%	13%	15%	5%	5%
Torilli [45]	1990-2009	297	Ca 42.0%	3%	Ca 36 months	76%	49%	38%	46%	28%	18%
Ruzzenetl [15]	1991-2007	17	-	-	10 months	-	-	20%	-	-	-
Chang [13]	1991-2006	160	-	2.7%	Ca 22 months	58%	34%	29%	32%	24%	22%
Roayaie [29]	1992-2010	165	-	7.3%	13 months	52%	22%	14%	40%	20%	18%
Daisuke [16]	1992-2008	45	22.2%	0%	20 months	70%	37%	22%	30%	21%	0
Ikai [46]	1992-2003	976	-	-	Ca 12 months	71%	23%	11%	48%	16%	4%
Hiroyki [37]	1990-2009	34	44%	2.9%	-	-	-	20%	-	-	-
Inoue [21]	1995-2006	49	-	0%	Ca 34 months	60%	45%	40%	35%	30%	20%
Liang [23]	2001-2005	53	-	1.9%	6 months	23.4%	5.8%	-	8.4%	4.2%	-
Peng [27]	2002-2007	201	4%	0.5%	20 months	42%	14.10%	11.1%	23%	8.5%	2.8%
Liu [34]	2002-2012	247	-	-	64 months	85%	68%	61%	-	-	-
Zhou [48]	2003-2010	121	-	-	10 months	47%	20%	-	-	-	-
Tang [31]	2006-2008	186	36%	23.7%	10 months	40%	14%	-	32%	6%	-
Chen [17]	2006-2008	88	19.3%	4.5%	9 months	31%	15%	-	-	-	-
Wu [33]	1990-1998	15	40%	0%	Ca 24 months	87%	45%	26%	54%	32%	21%
Kim [41]	2006-2010	83	-	-	25 months	68.6%	41.6%	-	30.6%	21.2%	-
Liu [40]	2000-2009	65	32.3%	-	17 months	80.1%	0%	0%	73.3%	0%	0%
Lin [42]	1996-2000	21	42.90%	4.80%	21 months	Ca 70%	Ca 21%	Ca 9%	-	-	-
	2001-2005	47	44.70%	4.30%	36 months	Ca 71%	Ca 58%	Ca 28%	-	-	-
Fan [19]	1997-2002	84	-	-	15 months	39%	16%	-	-	-	-
Huang [20]	1998-2008	116	30.20%	3.40%	Ca 21 months	71%	23%	11%	48%	16%	4%
Peng [26]	1997-2001	63	-	1.90%	7.8 months	18%	15%	2%	-	-	-
Zhong [14]	2000-2007	248	27.00%	4.40%	-	81%	46%	20%	55%	29%	20%
Shi [30]	2001-2003	406	32.80%	0.20%	-	34%	13%	-	13%	5%	-
Zheng [36]	2000-2008	96	35.40%	1.00%	-	86.5%	60.4%	33.3%	-	-	-
Kokudo [39]	2000-2007	CPA 1877	-	-	2.87 years	74.8%	49.1%	39.1%	-	-	-
		CPB 216	-	-	1.44 years	61.3%	35.2%	25.6%	-	-	-
Chen [18]	1990-2003	286/152^*^	15.5%/21%^*^	0%/2.6%^*^	18.8 months/10.1 months^*^	58.7%/39.5%^*^	22.7%/5.7%^*^	18.1%/0%^*^	-	-	-
Xu [49]	2008-2012	40/16^*^	20%/25%^*^	0%/6.25%^*^	-	62.3%/31.5%^*^	16.1%/0%^*^	5.2%/0%^*^	-	-	-
Kondo [47]	1990-2008	43/5^*^	4.7%/60%^*^	-	398 days/248 days^*^	-	-	-	-	-	-

ca.=approximately (for data estimated from published graphs), DFS=disease-free survival, OS=overall survival, CPA= Child-Pugh A, CPB=Child-Pugh B.

^*^The first value refers to patients in whom portal vein tumor thrombosis located within the first branch of the main portal vein. The second value refers to patients in whom portal vein tumor thrombus extended into the main portal vein.

### Surgical resection vs. non-surgical treatment for HCC with accross degrees of PVTT

The location and extent of a PVTT plays an independent prognostic role in determining surgical outcomes. A recently published Japanese nationwide survey [39] of 6474 patients from a number of medical centers showed that surgical resection is associated with a longer survival outcome than non-surgical treatment, as long as the PVTT is limited to the first-order branch (VP1–3 PVTT). In a subgroup analysis, the results demonstrated that surgical resection achieved a longer survival period than non-surgical resection in patients with both Child–Pugh A and Child–Pugh B liver function. Results after propensity score matching in patients with Child–Pugh A liver function further confirm that the 1-, 3-, and 5-year cumulative survival rates after surgical resection were significant higher than those obtained for non-surgical treatment. Moreover, other subgroup analyses after propensity score matching demonstrated that the survival rate after surgical resection was significant better than that after non-surgical treatment, regardless of age (older or younger than 70), and etiology of underlying liver diseases, including the presence or absence of viral infection, positive or negative serum α-fetoprotein, and single or multiple tumors. However, surgical resection did not achieve a better survival outcome than non-surgical treatment in patients with VP4 PVTT. Likewise, a number of reports from mainland China and Taiwan concerning all degrees of PVTT revealed concordant results. Studies from Liu [34], Peng [27], and Wang [35] compared the therapeutic effects of surgical resection and TACE for patients with HCC and PVTT using Cheng's PVTT classification. Their results after propensity scores matching were completely consistent: surgical resection achieved higher 1-, 3-, and 5-year survival rates than TACE for patients with HCC with Cheng's I–II PVTT, while the corresponding survival outcomes were not significant in patients with Cheng's III–IV PVTT. Studies on survival outcomes after surgical resection versus TACE are shown in Table 3. In a meta-analysis, [57] stratified studies revealed that patients with I–II type PVTT who underwent surgical resection had a better survival outcome than those who underwent TACE. However, for patients with III–IV PVTT, TACE was more suitable.

**Table 3:** Studies on survival outcomes of surgical resection versus non-surgical treatment HCC with all degrees of PVTT

Studies	Type of PVTT	Treatment approaches (numbers of recruited patients)	OS rates (%)				
			Median survival periods	1-yr	3-yr	5-yr	P value
Peng [27] (2012)	I-IV	Hepatectomy+thrombectomy (201)	20.0 ± 1.8 months	42.0%	14.1%	1.1%	<0.001
(1:2 PSM) (China)		TACE (402)	13.1 ± 0.6 months	37.8%	7.3%	0.5%	
	I	Hepatectomy+thrombectomy (27)	-	81.5%	1.2%	7.9%	<0.001
		TACE (54)	-	41.1%	8.9%	3.6%	
	II	Hepatectomy+thrombectomy (201)	-	46.3%	7.2%	7.2%	0.002
		TACE (402)	-	37.9%	6.0%	0%	
	III	Hepatectomy+thrombectomy (201)	-	32.5%	3.6%	3.6%	0.541
		TACE (402)	-	36.1%	4.2%	0%	0.371
	IV	Hepatectomy+thrombectomy (201)	-	21.7%	0%	0%	
		TACE (402)	-	30.4%	4.2%	0%	
Wang [35] (2016)	I	SR (122)	14.7 months	57.4%	21.0%	10.0%	0.001
(PSM) (China)		TACE (45)	8.69 months	40.0%	7.4%	0%	
		SR (50)	15.11 months	60.0%	23.8%	17.1%	0.039
		TACE-Sor (21)	12.01 months	52.4%	0%	0%	
	II	SR (187)	12.11 months	50.8%	22.3%	13.3%	0.000
		TACE (187)	5.279 months	25.1%	5.3%	4.5%	
		SR (80)	18.1 months	61.3%	38.3%	18.2%	0.001
		TACE-Sor (32)	8.92 months	30.3%	0%	0%	
		SR (131)	15.29 months	59.5%	28.3%	11.9%	0.046
		TACE-RT (47)	11.7 months	48.9%	17.2%	0%	
	III	SR (171)	6.17 months	36.3%	8.2%	2.6%	0.150
		TACE (171)	5.16 months	28.1%	8.7%	5.7%	
		SR (76)	5.42 months	31.6%	4.9%	-	0.166
		TACE-Sor (31)	7.96 months	28.7%	20%	-	
		SR (50)	5.42 months	34.0%	11.4%	-	0.401
		TACE-RT (50)	7.96 months	40.0%	15.4%	-	
Liu [34] (2014)	I-II	SR (108)	64.0 months	84.0%	69.0%	59.0%	0.004
(PSM) (Taiwan)		TACE (108)	32.0 months	71.0%	50.0%	35.0%	
Zhong [14] (2014)(PSM) (China)	I-IV	SR (248)	-	68.0%	46.0%	20.0%	
Kokudo [39] (2011)	VP1-4	Child-Pugh ALR (1877)	2.87 years	74.8%	49.1%	39.1%	<0.0001
(Japan nationwide survey)		non-LR^*^ (2512)	1.10 years	53.1%	25.3%	16.0%	
		LR(216)	1.44 years	36.3%	35.2%	25.6%	<0.0001
		non-LR(1869)	0.48 years	32.2%	13%	7.9%	
Zheng [36] (2011)	I-IV	Hepatectomy (96)	-	86.5%	60.4%	33.3%	0.021
(China)		TACE (134)	-	77.6%	47.8%	20.52%	
Zhou [48] (2011)	I-III	Hepatectomy+thrombectomy (23)	10.0 months	47%	22%	-	<0.05
(China)		Hepatectomy+thrombectomy+portal vein Chemobiotherapy (31)	16.0 months	70%	20%	-	
		TACE (10)	7.0 months	10%	0%	-	
		Conservative (30)	3.0 months	12%	4%	-	
Fan [19] (2005)	I-III	Surgical resection (24)	10.1 months	22.7%	0%		<0.001
(China)		surgical resection + postoperative chemotherapy (84)	15.1 months	39.3%	15.6%		
		Chemotherapy (n=53)	7.3 months	11.8%	0%		
		Conservative treatment (18)	3.6 months	0%	0%		

SR=surgical resection, LR=liver resection, RT=radiotherapy, PSM= propensity score matching.

^*^Non-LR managements included TACE or chemotherapy or transaterial chemoinfusion or ablation or palliative support care.

Although surgical resection is associated with an inevitable risk of hospital mortality, complications, and recurrence, this aggressive approach is effective and safe for selected patients with HCC and PVTT who have a promising survival outcome, as long as the PVTT is limited to the first-order branch (Cheng's I–II or VP1–3 PVTT). By contrast, when the PVTT extends to the contralateral bifurcations or beyond the main portal vein, surgical resection fails to provide a better survival benefit than non-surgical treatment and patients might have lose the optimal opportunity of surgical treatment (Cheng's III–IV or VP4 PVTT).

### En-bloc resection vs. thrombectomy

At present, advanced surgical techniques are feasible to remove PVTT completely. If the tumor thrombus lies beyond the resection line, extends to the contralateral bifurcation or the main truck, or invades the portal vein wall, it is not possible to remove the PVTT together with the primary tumor. An additional thrombectomy or en-bloc resection with or without portal vein reconstruction should be performed [16, 33, 38, 43]. Studies from Daisuke [16], Kenneth [38], Li, [43] and Wu [33] compared the surgical procedures, complication, survival outcomes, and recurrence between the approaches of thrombectomy and en-bloc resection. Daisuke [16] and Kenneth [38] showed that hepatectomy plus thrombectomy or en-bloc resection may provide a comparable survival benefit for patients with VP4 or VP3 PVTT. Li [43] showed that both overall survival and disease-free survival at 1, 3, and 5 years were not significantly different for patients with PVTT within the resection line treated with en-bloc resection and patients with PVTT beyond the resection area treated with thrombectomy. Wu [33] demonstrated that both disease-free survival and overall survival at 5 years were not significant reduced in patients with PVTT extended to the bifurcation of portal vein if en-bloc resection was performed. In addition, Wu's results [33] revealed that these two approaches are not significant different in terms of morbidity and in-hospital mortality. The decision of such surgical manipulations could be based on various factors, including the surgeon's expertise in portal vein resection and reconstruction, as well as the nature of the thrombus. Details of surgical procedures and outcomes between thrombectomy and en-bloc resection are shown in Table 4.

**Table 4:** Surgical outcomes of en bloc resection or thrombectomy for HCC with VP4 PVTT versus Hepatectomy for HCC with VP3 PVTT

Studies	Enrollment period	Type of PVTT and total patients	Surgical approaches	Morbidity		Hospital mortality		OS					DFS				
				Rates	P value	Rates	P value	Median survival period	1-yr	3-yr	5-yr	P value	Median survival period	1-yr	3-yr	5-yr	P value
Daisuke [16] Japan	2009	VP3 (n=26)	Hepatectomy	23.1%	1.00	0%	-	18 months	72%	35.3%	21.2%	0.821	-	-	-	-	-
		VP4(n=19)	Hepatectomy+ thrombectomy	21.1%		0%		28 months									
Wu [33] China	2000	PVTT extension to portal vein bifurcation (15)	Hepatectomy+ portal vein partial resection and reconstraction	40%	0.11	0%	>0.99	-	-	-	26.4%	0.33	-	-	-	21.6%	0.19
		PVTT limited to single branch of portal vein(97)	Hepatectomy	20.6%		3.1%		-	-	-	28.5%		-	-	-	20.4%	
Kenneth [38]	2013	VP3 (n=71)	Hepatectomy	31.9%	0.079	2.8%	0.44	10.91 months	45.8%	22.7%	14.3 %	0.962	4.21 months	24.3%	14.3%	10.7%	0.363
Japan		VP4 (n=7) both branches	Hepatectomy+ thrombectomy	71.4%		0%		9.4 months	50%	12.5%	12.5%		3.78 months	14.3%	14.3%	14.3%	
		VP4 (n=10) both branches	En bloc resection with PV reconstruction	50%		10%		8.58 months	28.6 %	14.3 %	11.2%		1.51 months	0%	0%	0%	
Li [43]	2015	PVTT not extending to the superior mesenteric vein (38) and (39)	En bloc resection	18.4%	0.817	0%	-	14.3 months	58.5%	32.9%	29.2%	0.047	3.7 months	32.5%	15.2%	15.2%	0.191
Taipei			Thrombectomy	20.5%		0%		10.4 months	42.6%	11.4%	5.7%		2.7 months	15.4%	5.1%	5.1%	

### Elevation of life quality after surgical resection

Removal of PVTT has the advantage of reducing the tumor burden, relieving portal vein hypertension and its related complications, and improving the recovery of liver function, leading to an enhancement of life quality [19]. A study by Liu [40] conducted Functional Assessment of Cancer Therapy-Hepatobiliary (FACT-HEP) to compare the life qualities between patients with HCC and PVTT who underwent surgical resection and those who chemotherapy. This assessment includes 45 items, divided into 5 dimensions: physical well-being (7 items), social or family well-being (7 items), emotional well-being (6 items), functional well-being (7 items), and hepatobiliary function (18 items). Respondents use a five-point Likert scale, ranging from 0 (not at all) to 4 (very much), with higher scores reflecting better health-related life quality. The questionnaires were completed before surgery or chemotherapy and 3, 6, 9, and 12 months after surgery or chemotherapy. If the survival period was shorter than 12 months, the records were maintained until the last score. Liu [40] reported that the FACT-HEP scores in the surgery group were significant higher than the scores in the control group. According to this survey, [40] surgical resection is more effective in elevating life quality than is non-surgical resection. However, the benefit of surgical resection in enhancing life quality remains to be further evaluated by objective and subjective measurements, including FACT-HEP scores, serological tests, and imaging examinations.

## MULTIDISCIPLINARY DIAGNOSIS AND TREATMENT TO IMPROVE THE EFFICACY AND SAFETY OF SURGICAL RESECTION

### Accurate preoperative assessment of anatomic features and future hepatic function reserve

With development of advanced imaging examinations, the efficacy and safety of surgical resection for HCC and PVTT have been greatly improved. As compared to conventional imaging detection, novel imaging techniques could provide better understanding of anatomic features of tumor and prediction of remnant liver function reserve, which is eligible for surgeons to make delicate plans for surgical procedure.

Complete resection of the primary tumor and PVTT and sufficient liver function reserves are essential for surgical planning. Initially, PVTTs tend to grow infiltratively; thus, the surgical procedure should attempt to achieve an eradicative resection of the primary tumor with an adequate resection margin, and a complete removal of the tumor thrombus according to the tumor extent and the severity of portal vein invasion [18, 21, 30, 58, 59]. In a retrospective study of 381 patients, Zhou [44] reported that a surgical resection margin of at least 5 mm is an independent prognostic factor. Normally, evaluation of such a surgical procedure is made using conventional two-dimensional multislice computed tomography (MSCT). By contrast, three-dimensional imaging and reconstruction, taking advantage of stereoscopic 360° imaging of the tissues would decrease the sampling and observing error resulting from the two-dimensional images [60]. Thus, three-dimensional imaging and reconstruction might provide a better understanding of anatomic features of tumor, including the tumor size, location, relationships with adjacent vessels and tissues, which is feasible for surgeon to make precise plan for surgical procedure. Some studies proved that three-dimensional imaging and reconstruction is useful in preoperative planning for surgical resection by providing precise information on the tumor-free margin [61–64]. Moreover, recently this advanced imaging examination had been used to detected PVTT and simulated the surgical resection of HCC and PVTT. A new study reported by Wei and *et al.* [65] firstly demonstrated that three-dimensional imaging and reconstruction provided a more precise detection of the PVTT and a more accurate simulation of the operative procedure as compared with 2D MSCT. In this study, three-dimensional imaging had a relative higher accuracy of prediction of the PVTT classification than 2D MSCT (87.1% vs. 81.4%); in addition, there was a significant correlation between estimated and actual volumes of the resected specimens and resection margins. The difference between the estimated volume and the actual volume was 66.2 ± 52.5 ml (*P*<0.01), while the difference between the predicted and the actual margins was 2.8 ± 2.1 mm (*P*<0.01). According to follow-up data, three-dimensional reconstruction significantly improved disease-free survival (6, 12, 18, and 24 month disease-free survival rates were 56.1%, 20.1%, 16.5%, and 12.3% for three-dimensional imaging but 33.3%, 11.1%, 8.3%, and 5.6% for multislice computed tomography, *P*=0.022) and overall survival (6, 12, 18, and 24 month overall survival rates were 90.2%, 73.5%, 54%, and 40% for three-dimensional imaging but 88.3%, 59%, 27.1% and 18% for multislice computed tomography, *P* = 0.020). Moreover, patients with a type I–II PVTT had a significantly higher rate of en-bloc resection or thrombectomy (*P*=0.025), a shorter operation time (167.4 ± 42.6 min vs. 200.2 ± 71.3 min, *P*=0.026) and a shorter hilar clamp time (16.9 ± 5.2 min vs. 19.6 ± 4.7 min, *P*=0.025) if they were examined using three-dimensional reconstruction than if they were examined using two-dimensional multislice computed tomography. In addition, it is now possible to use three-dimensional printing after three-dimensional imaging scanning and reconstruction to create an artificial model, which is helpful to improve the understanding of the anatomic characteristics and relationships of the primary tumor, non-invaded vessels, non-tumor liver tissues, severity of vascular invasion, and PVTT. Therefore, surgeons could design delicate surgical plans and accurate simulations of the operative procedure to enhance the efficacy of surgical resection for both primary tumor and PVTT.

Besides, postoperative liver failure is a major risk factor of hospital mortality, especially for patients with liver cirrhosis and continuous deterioration of liver function by active hepatitis. Preoperative accurate assessment of sufficient future liver function reserve is important in ensuring the safety of surgical resection [66, 67]. Conventionally, the indocyanine green clearance test is used to evaluate functional hepatocytes to predict remnant liver function, while computed tomography volumetry provides anatomic information on remnant liver volume but not functional volume [66, 67]. However, a single liver function test or imaging volumetry are not sufficiently accurate by themselves in predicting the liver function status and complications after resection. A combination of such established non-invasive approaches may provide a better prediction of remnant liver function. By contrast, novel assessments, such as 99mTc-galactosyl serum albumin scintigraphy combined with computed tomography volumetry [66, 67] and three-dimensional reconstruction, and gadolinium-ethoxybenzyl-diethylenetriamine pentaacetic acid enhanced MRI [68] are better quantitative measures of future liver functional volume and regeneration ability and can give a more accurate prediction of liver-related morbidity after hepatic resection in patients with HCC. These advanced preoperative assessments are convenient for surgeons to determine suitable candidates for resection and helpful to improve the safety of surgical resection.

## REGIONAL ADJUVANT THERAPIES

### Postoperative TACE to suppress early recurrence

Portal vein vascular invasion to residual tumors and promote intrahepatic metastasis, which has been well defined and accepted as a major mechanism contributing to early recurrence (2 years after resection), leading to poor postoperative survival [5–9]. Recent findings indicate that microvascular invasion is another independent risk factor for early recurrence in single HCC without macrovascular invasion [69–72]. Moreover, compression and crushing of tissue during the operative process might create new intrahepatic metastases. Postoperative TACE involving a combination of occlusion of feeding arteries and locally administered chemotherapy has been widely used as a common adjuvant intervention combined with surgical resection. Injected lipidol via a TACE procedure could selectively accumulate in the invisible metastatic HCC to block most of its nutrient vessels when delivered intra-arterially, while acting as a carrier for anticancer drugs, allowing sustainable chemotherapeutic killing of the HCC cells [73–75]. One meta-analysis compared the outcomes of surgical resection combined with postoperative TACE versus surgical resection alone [76]. Subgroup analyses of vascular invasion demonstrated that both disease-free survival and overall survival were statistically significantly improved in hepatic resection for patients who underwent postoperative TACE, compared with their counterparts who underwent hepatic resection without postoperative TACE. Three randomized controlled trials reported by Li, [77] Peng, [78] and Zhong [79] were consistent in demonstrating postoperative TACE as useful in improving both disease-free survival and overall survival for patients with HCC and PVTT after surgical resection. Likewise, retrospective studies reported by Cheng [80] and Ren [81] proved that postoperative TACE following surgical resection achieved a better survival outcome than surgical resection alone for patients with HCC and PVTT, with a preventative role of restraining early recurrent tumorigenesis, especially within the first 6 months after surgical resection [82]. In these two retrospective studies residual tumors was identified as the independent risk factor of very early recurrence (within the first 6 months after surgical resection), and adjuvant TACE was the significant prognostic factor for patients with high risks of residual tumor. Thus, according to their therapeutic recommendation TACE should be used in patients with high risks of residual tumors but not in patients with low risks of residual tumors. Studies of the disease-free survival and overall survival outcomes after surgical resection plus postoperative TACE versus surgical resection along are summarized in Table 5.

**Table 5:** Studies on multidisciplinary diagnosis and adjuvant treatments to improve efficacy or safety of surgical resection

Study (years)	Treatment approaches (numbers of recruited patients)	OS					DFS				
		Median survival periods	1-yr	3-yr	5-yr	*P* value	Median survival periods	1-yr	3-yr	5-yr	*P* value
**Postoperative TACE**											
Peng [78] (2009) (RCT)	Hepatectomy plus TACE (51)	13 months	50.9%	33.8%	21.5%	0.0094	-	-	-	-	
	Hepatectomy (53)	9 months	33.3%	17.0%	8.5%		-	-	-	-	
Zhong [79] (2009) (RCT)	Hepatectomy + TACE (57)	23 months	80.7%	33.3%	22.8%	0.0048	6 months	29.7%	9.3%	9.3%	0.004
	Hepatectomy (58)	14 months	56.5%	19.4%	17.5%		4 months	14.0%	3.5%	1.7%	
Ren [81] (2004)^*^	Hepatectomy + TACE (987)	Non-available	89.6%	61.28%	44.36%	0.0216	-	-	-	-	
	Hepatectomy alone (643)	Non-available	69.95%	49.86%	37.4%		-	-	-	-	
Cheng [80] (2005)	Hepatectomy + TACE (987)	-	-	-	-	-	77.8% (0.5-yr)	21.99%			<0.0001
	hepatectomy alone (643)	-	-	-	-	-	38.4%	25.3%			
Fan [19](2005)	surgical resection+ postoperative chemotherapy (84)	15.1 months	39.3%	15.6%	-	<0.001	-	-	-	-	-
	Surgical resection (24)	10.5 months	22.7%	0%	-		-	-	-	-	-
**Preoperative radiotherapy**											
Li [86](2016)	3DCRT + SR (45)	-	69.0%	20.4% (2-yr)	-	<0.01					
	SR (50)	-	35.6%	0%(2-yr)	-						
Toshiya [101](2007)	Preoperative radiotherapy + hepatectomy (15)	-	86.2%	43.5%	34.8%	0.0359					
	hepatectomy (28)	-	39.0%	13.1%	13.1%						

RCT=randomized control trials, 3DCRT=three-dimensional conformal radiotherapy, SR=surgical resection.

^*^Including primary tumor >5cm, pulti-nodulars, and macro-vascular invasion.

Further, tiny recurrent lesions usually escape detection or present with untypical imaging characteristics of malignant neoplasm using traditional imaging techniques at an early stage. Uncertainty in the detection of small HCC lesions usually leads to delays in patients receiving subsequent interventions for recurrent HCC, making it difficult for them to be treated at the optimal time. Lipidol computed tomography is more reliable than traditional imaging techniques in discovering small HCC lesions, [82] which usually present as a stable tumor staining after lipidol injection, while normal liver parenchyma remains clear, within a week. Clearly with this situation, TACE potentially plays an equal monitoring role to lipidol computed tomography in enhancing the efficacy of imaging scans in monitoring small recurrent nodules. The HCC-specific uptake of lipidol would be discovered by computed tomography within 3–4 weeks after lipidol embolization. Thus, confirmatory diagnosis of small recurrence at an early stage may provide an optimal opportunity of subsequent interventions.

At present, some guidelines in the Asia-Pacific region approve postoperative TACE as an effective adjuvant intervention following surgical resection to improve survival and is clearly recommended for patients with HCC and PVTT after surgical resection, with spontaneous preventative and therapeutic roles, and a potential detective role for recurrent HCC.

### Preoperative adjuvant radiotherapy

In the past, radiotherapy was seldom used in HCC, as it is a radio-resistant squamous cell carcinoma [83]. It is difficult to acquire a complete response for primary HCC, even treated with high-dose irradiation. Meanwhile, high-dose irradiation usually induces radiation injury to the non-tumorous liver as well as serious complications, such as leucopenia or thrombocytopenia [83–85]. Recently, developments of novel external three-dimensional conformal radiotherapy (3DCRT) [86] and selective internal radiotherapy (SIRT) with yttrium-90 microspheres [87–99] show potential for concentrating irradiation to the primary tumor and PVTT while at the same time sparing normal liver tissues from irradiation to minimize irradiative injury to non-tumor liver volume. These approaches might downstage the unresectable primary tumor and PVTT, allowing salvage resection, while improving survival outcomes after surgical resection.

A large series of studies has proved that three-dimensional conformal radiotherapy (3DCRT) for unresectable and advanced HCC is effective in controlling disease progression with accepted toxicity. Recently, a study reported by Li *et al.* [86] firstly demonstrated that a novel 3DCRT three-dimensional conformal radiotherapy with a low-dose external irradiation to the tumor administered over a long duration was effective in downstaging both primary HCC and PVTT and avoiding radiation-induced liver disease for allowing salvage resection. In this study, during the radiotherapy, a daily fraction of 300 cGy was administered for 6 consecutive days, giving a total irradiation dose of 1800 cGy for patients. A 50–80% isodose was prescribed for the distribution areas. Downstage reduction was considered effective if the volume of the primary tumor or the extent of the PVTT decreased by at least 30%. Interestingly, in this study, PVTTs presented a better radio-response rate than HCC (PVTT vs. HCC response rate: 27% vs. 13%). Also, they revealed that this preoperative novel neoadjuvant radiotherapy was effective in improving overall survival and decrease recurrence rates at 6 and 12 months after surgical resection and improving cumulative survival rates during the first 2 years (the 6 month and 12 month recurrence rates were 49% vs. 77% for the patients underwent neoadjuvant radiotherapy followed by surgical resection, and 88.7% and 97.7% for the patients only underwent surgical resection, *P*<0.01; meanwhile, the 1- and 2- year overall survival of three-dimensional conformal radiotherapy followed surgical resection versus surgical resection alone were 69% vs. 20.4%, and 35.6% vs. 0%, *P*<0.01;). Moreover, this study indicates that “downstaging of PVTT” could shorten operation time and decrease bleeding, and the likelihood of squeezing or fragmenting the tumor thrombus by thrombectomy or en-bloc resection following portal vein reconstruction during the operation, thus elevating the safety of surgical resection and minimizing the chance of residual tumor cells spreading.

Transarterial radioembolization (TARE) with yttrium-90 glass as a commonly used selective internal radiotherapy (SIRT) involves the transarterial administration of therapeutic doses of radiation to the liver tumor. During the radioembolization procedure, a radioactive microsphere is selectively injected into the hepatic artery or its branches to deliver intense local radiation to the tumor while non-tumor liver tissues and the rest of the patient's body are protected from irradiative injury. According to the reports, the disease control rates ranged from 58% to 73%, with tumor responses to irradiation of complete response, partial response, or stable disease. Tolerance was good; all reported adverse events were grade 3 or less. The most frequent adverse event is a transient fatigue syndrome. Studies on radiotherapies to downstage HCC and PVTT for allowing salvage resection are summarized in Table 6. A study from Law [87] evaluated strategies to downstage unresectable HCC for allowing salvage resection; this study proved that TARE with yttrium-90 is an effective and safe approach for combination with systematic chemotherapy. In this study, 7 of 49 patients had unresectable HCC and PVTT involved the main portal vein. After tumor downstaging and salvage resection, a promising overall survival rate of 57% was achieved at 5 years. Pracht [90] demonstrated that TARE with yttrium-90 was eligible for downstaging the PVTT, allowing salvage resection for selected patients with complete or partial tumor response. In this study, [90] 4 patients (22%) after tumor downstage were eligible for surgery. Two of these met the liver transplantation criteria [100] and the other 2 patients were eligible for surgical resection. After salvage resection, the patients showed a progression-free survival of 13.8 and 10.5 months, and an overall survival of 14.8 and 12.5 months.

**Table 6:** Studies on efficacy and safety of radiotherapy to downstage the HCC and PVTT for allowing salvage resection

Studies	Population of downstage for resection	Radiotherapy approaches	Response (CR/PR/SD/PD) numbers	Response rates	Disease-control rates	Adverse events
**Li** [78] (2016)	6/45	3DCRT	For PVTTCR=0/PR=6^^^/SD=31/PD=2For HCCCR=0/PR=6/SD=35/PD=4	For PVTT 27%For HCC 13%	For PVTT 94.9%For HCC 90.9%	no severe adverse effect relating to radiotherapy, only 2 patients with deterioration in liver function developed contraindications to partial hepatectomy
**Pracht** [83] (2013)	4/18 ^*^	TARE with Yttrium-90	CR=2/PR=13/SD=1/PD=2	83.3%	88.9%	Tolerance was good and Adverse effects were Grade 3 or less on the CTCAEv3.0 scale. No deaths attributed to the treatment
**Toshiy** [97] (2007)	15/15	External regional RT for PVTT	Only for PVTTCR=8/PR=7	-	-	No apparent radiation-induced complications and radiation hepatitis were observed except one patients suffered severe nausea and vomiting.

3DCRT= three-dimensional conformal radiotherapy, CR= complete response, PR=partial response, SD=stable disease, PD=progressive disease, TARE= transarterial radioembolization.

^*^2 of them to be within liver transplantation criteria, and the other two patients are feasible for surgical resection.

^^^Cheng's type III PVTT downstaged to Cheng's type III PVTT.

Other approaches of preoperative neoadjuvant radiotherapy have also been reported. Toshiya [101] compared survival outcomes in patients who underwent preoperative neoadjuvant regional external radiotherapy followed by surgical resection with patients who underwent surgical resection alone. In this study, external radiotherapy only targeted the PVTT, not the whole gross tumor. During the radiotherapy procedure, 6 or 10 MV X-ray means were used. Irradiative doses were 30 Gy in 10 fractions for 13 patients and 36 Gy in 12 fractions for 12 patients; the treatment period was 15–20 days. No apparent radiation-induced complications and no radiation hepatitis were observed, except that one patient suffered severe nausea and vomiting. Eight of 15 patients showed a completely necrosed tumor thrombus after external and regional irradiation, and the overall survival rates at 1, 3, and 5 years were significant greater for patients undergoing radiotherapy plus surgical resection than for patients undergoing surgical resection only (median survival periods were 1.63 vs. 0.76 years; overall survival at 1, 3, and 5 years was 86.2% vs. 39.0%, 43.5% vs. 13.1%, and 34.8% vs. 13.1%, *P*=0.0359). Studies on the disease-free survival and overall survival outcomes after preoperative neoadjuvant radiotherapy followed by surgical resection versus surgical resection alone are summarized in Table 5.

Therefore, neoadjuvant radiotherapy followed by surgical resection is expected to improve postoperative survival outcomes. However, the efficacy and safety of radiotherapy as a preoperative adjuvant therapy require further validation in prospective randomized clinical trials. Moreover, at present, there is no uniform radiotherapy approach or standard dosage for adjuvant external or internal radiation therapies.

## SYSTEMATIC THERAPIES FOR PREVENTION OF RECURRENCE

As an oral administration of anti-angiogenesis, [102] sorafenib has been defined as the standard treatment for patients with advanced HCC. There is a hypothesis that this molecular target medicine acts as a systematic adjuvant therapy with surgical resection to prevent recurrence and improve survival. Despite some animal studies indicating a positive effect of sorafenib in inhibiting intrahepatic tumor recurrence and abdominal metastasis after liver cancer resection and consequent improvement of survival in a nude mouse model, [103, 104] unfortunately the STORM trial [105]—as a phase III, randomized, double-blind, placebo-control clinical trial—presents a negative effect of sorafenib in enhancing recurrence-free survival, time to recurrence, and overall survival in patients with HCC. Thus, sorafenib has not been strongly recommended for patients with HCC after surgical resection.

Conventionally, systematic cytotoxic chemotherapy presents significant toxicity, impaired liver function, and portal hypertension, which frequently occur in patients with PVTT [67]. Currently systematic chemotherapy is not used as an adjuvant therapy with surgical resection to prevent recurrence. However, since postoperative TACE as a local chemo-administration is unable to prevent extrahepatic tumor recurrence and sorafenib as a systematic therapy has a negative effect in failing to prevent either intrahepatic or extrahepatic tumor recurrence, future novel systematic chemotherapy regimens to suppress recurrence are worth exploring.

In addition, antiviral therapy is routinely recommended to minimize the continuous impairment of liver function by hepatitis and to prevent tumor recurrence.

## UPDATING OF GUIDELINES OF MULTIDISCIPLINARY TREATMENTS FOR HCC IN ASIA-PACIFIC REGION

At present, based on clinical characteristics, long-term and short-term therapeutic outcomes, and the safety of intervention, several guidelines for multidisciplinary treatments for HCC in the Asia-Pacific region, including mainland China, Hong Kong, and Japan have been updated to identify surgical treatment as a feasible option for selected patients with HCC and PVTT. A new published “*Chinese expert consensus on multidisciplinary diagnosis and treatment of hepatocellular carcinoma with portal vein tumor thrombus: 2016 edition*” [50] approves surgical resection as eligible for primary resectable HCC and Cheng's I–III type PVTT and recommends that adjuvant therapies, such as preoperative radiotherapy or postoperative TACE, should be added. The “*Hong Kong Consensus Recommendations on the Management of Hepatocellular Carcinoma*”, [51] published in 2015, indicates that intrahepatic vascular invasion is not an absolute contradiction for surgical resection in selected patients with Child–Pugh A liver function and tumor size no larger than 5 cm. Similarly, a “*2014 update JSH Consensus-based Clinical Practice Guildelines for the Management of Hepatocellular Carcinoma*” by the liver cancer study group of Japan [52] supports surgical resection as feasible for selected patients with HCC, with VP1–3 type PVTT and Child–Pugh A liver function. Moreover, in a consensus statement “*surgery for intermediate and advanced hepatocellular carcinoma: a consensus report from the 5^th^ Asia-Pacific primary liver cancer expert meeting (APPLE 2014)*” [53] 10 top Asian experts from 10 institutions voted that portal venous invasion should not be defined as a contra-indication for surgical resection, the final votes for *yes* vs. *no* were 0:10. This meeting established a common expert consensus to identify PVTT as not an absolute contra-indication for surgical resection.

## CONCLUSIONS AND FURTHER EXPECTATIONS

### Current treatment status of surgical resection

Currently, advanced surgical techniques allow for complete resection of both primary tumor and PVTT. According to our analysis, surgical resection has achieved great improvements in prolonging survival periods while decreasing postoperative complications in past few years. In most centers in the Asia-Pacific region, PVTT is not identified as an absolute contra-indication of surgical resection. Surgical resection may be considered in selected patients with HCC and PVTT beyond the BCLC treatment algorithms, as long as the PVTT is only located in the segmental or an ipsilateral bifurcation portal vein but not extended to the contralateral bifurcation or the main truck, so long as the primary tumor is eligible for resection and remnant liver function is sufficient. However, patients with bad surgical performance and have impaired liver function may not benefit from surgical resection. In addition, surgical resection is associated with an inevitable risk of hospital mortality, morbidities and recurrence. In these situations, advanced multidisciplinary diagnosis and treatments are helpful in improving the efficacy and safety of surgical resection. These approaches include three-dimensional imaging and construction to provide an accurate preoperative assessment of anatomic features and remnant liver function reserve; preoperative neoadjuvant radiotherapy to downstage the primary tumor and PVTT; postoperative TACE to prevent and simultaneous monitor tumor recurrence; and subsequent treatment of recurrent tumors. Moreover, subsequent treatments for recurrent tumors support the value and necessity of surgical resection for patients with initial resectable tumor at the BCLC-C stage. A muliticenter study [106] enrolled patients with HCC of various ethnicities from eastern Asia, northern America, and southern Europe, indicated that some nonideal candidates may still potentially benefit from resection over other treatment modalities and the selection criteria for surgical resection may be modestly expanded. Therefore, surgical resection might be considered suitable for expanding patients with HCC and PVTT. When considering surgical resection as an option for these patients, clinicians must balance the potential benefits and adverse effects to determine the most appropriate management in individual patients.

So far, non-surgical treatments are unable to achieve a therapeutic effect comparable to surgical resection. Thus, surgical resection remains an irreplaceable strategy for selected patients with HCC with PVTT. However, surgical resection as a strategy for HCC with PVTT beyond the BCLC treatment algorithm remains in a dilemma: the major purpose of surgical resection is to prolong survival time and enhance life quality, instead of completely curing the malignant disease. Residual tumors and intrahepatic metastases that promote recurrence are still major obstacles for the treatment of HCC with PVTT. Future novel systematic therapies are worth exploring, to provide superior therapeutic effect with surgical treatment, while at the same time simplifying the management approach and reducing the burden of complications.

### Need for high-level evidence

Current evidence is mainly generated from retrospective studies or extrapolated from subgroup analyses of prospective clinical trials. At present, the issue of surgical resection for HCC with PVTT remains controversial. In spite of Bruix welcomes effective treatments beyond the BCLC treatment algorithms and added the statement of “*effective treatments with survival benefit*” to the new edition of BCLC treatment algorithms [107], current evidences are not strong enough to persuade western guideline to accepted surgical resection for advanced-stage HCC. Professor Bruix also stated that “in the absence of such clarifying information, the debate will continue.” [108]

In order to define the therapeutic effects and safety of surgical resection for HCC with different degrees of PVTT, randomized, double-blind, non-surgical treatment control trials could be persuasive. In practice, randomized controlled trials are difficult to carry out, with issues of ethical approval. The nearest Asia-Pacific primary liver cancer expert meeting (APPLE 2014) [53] voted to determine whether randomized controlled trials comparing surgical resection and sorafenib are still necessary for advanced-stage (BCLC-C) HCC. Four of 10 top experts insisted that randomized controlled trials remain irreplaceable, while the remaining 6 experts proposed a different option to support well-designed nonrandomized control trials as eligible to provide comparable evidence to randomized controlled trials. The controversy surrounding the necessity of randomized controlled trials remains among Asian experts. This issue should be the end of evidence-based medicine. No matter randomized controlled trials or nonrandomized controlled trials, well-designed perspective, larger size, and cohort studies for the generation of high-level evidence are necessary to evaluate the therapeutic effects and safety of surgical resection for HCC with different degrees of PVTT.

At the end of this review, we sincerely appreciated the BCLC treatment algorithm to provide a systematic staging and treatment algorithm for guiding the management of HCC. While at the same time, on the condition that when new evidences appears, treatment algorithm for HCC might be updated.
